# Genomic and Epigenetic Advances in Focal Cortical Dysplasia Types I and II: A Scoping Review

**DOI:** 10.3389/fnins.2020.580357

**Published:** 2021-01-22

**Authors:** Joana Jesus-Ribeiro, Luís Miguel Pires, João Daniel Melo, Ilda Patrícia Ribeiro, Olinda Rebelo, Francisco Sales, António Freire, Joana Barbosa Melo

**Affiliations:** ^1^Epilepsy and Sleep Monitoring Unit, Neurology Department, Coimbra University Hospital Center, Coimbra, Portugal; ^2^iCBR/CIMAGO, Faculty of Medicine, University of Coimbra, Coimbra, Portugal; ^3^Laboratory of Cytogenetics and Genomics, Faculty of Medicine, University of Coimbra, Coimbra, Portugal; ^4^CUF Coimbra Hospital, Coimbra, Portugal; ^5^Neuropathology Laboratory, Neurology Department, Coimbra University Hospital Center, Coimbra, Portugal; ^6^Faculty of Medicine, University of Coimbra, Coimbra, Portugal

**Keywords:** drug-resistant epilepsy, focal cortical dysplasia, genomics, mTOR pathway, epigenetics, DNA methylation, microRNAs, scoping review

## Abstract

**Introduction:** Focal cortical dysplasias (FCDs) are a group of malformations of cortical development that constitute a common cause of drug-resistant epilepsy, often subjected to neurosurgery, with a suboptimal long-term outcome. The past few years have witnessed a dramatic leap in our understanding of the molecular basis of FCD. This study aimed to provide an updated review on the genomic and epigenetic advances underlying FCD etiology, to understand a genotype–phenotype correlation and identify priorities to lead future translational research.

**Methods:** A scoping review of the literature was conducted, according to previously described methods. A comprehensive search strategy was applied in PubMed, Embase, and Web of Science from inception to 07 May 2020. References were screened based on title and abstract, and posteriorly full-text articles were assessed for inclusion according to eligibility criteria. Studies with novel gene variants or epigenetic regulatory mechanisms in patients that underwent epilepsy surgery, with histopathological diagnosis of FCD type I or II according to Palmini's or the ILAE classification system, were included. Data were extracted and summarized for an overview of evidence.

**Results:** Of 1,156 candidate papers, 39 met the study criteria and were included in this review. The advent of next-generation sequencing enabled the detection in resected FCD tissue of low-level brain somatic mutations that occurred during embryonic corticogenesis. The mammalian target of rapamycin (mTOR) signaling pathway, involved in neuronal growth and migration, is the key player in the pathogenesis of FCD II. Somatic gain-of-function variants in *MTOR* and its activators as well as germline, somatic, and second-hit mosaic loss-of-function variants in its related repressors have been reported. However, the genetic background of FCD type I remains elusive, with a pleomorphic repertoire of genes affected. DNA methylation and microRNAs were the two epigenetic mechanisms that proved to have a functional role in FCD and may represent molecular biomarkers.

**Conclusion:** Further research into the possible pathogenic causes of both FCD subtypes is required, incorporating single-cell DNA/RNA sequencing as well as methylome and proteomic analysis. The collected data call for an integrated clinicopathologic and molecular genetic diagnosis in current practice not only to improve diagnostic accuracy but also to guide the development of future targeted treatments.

## Introduction

**Figure 1:**
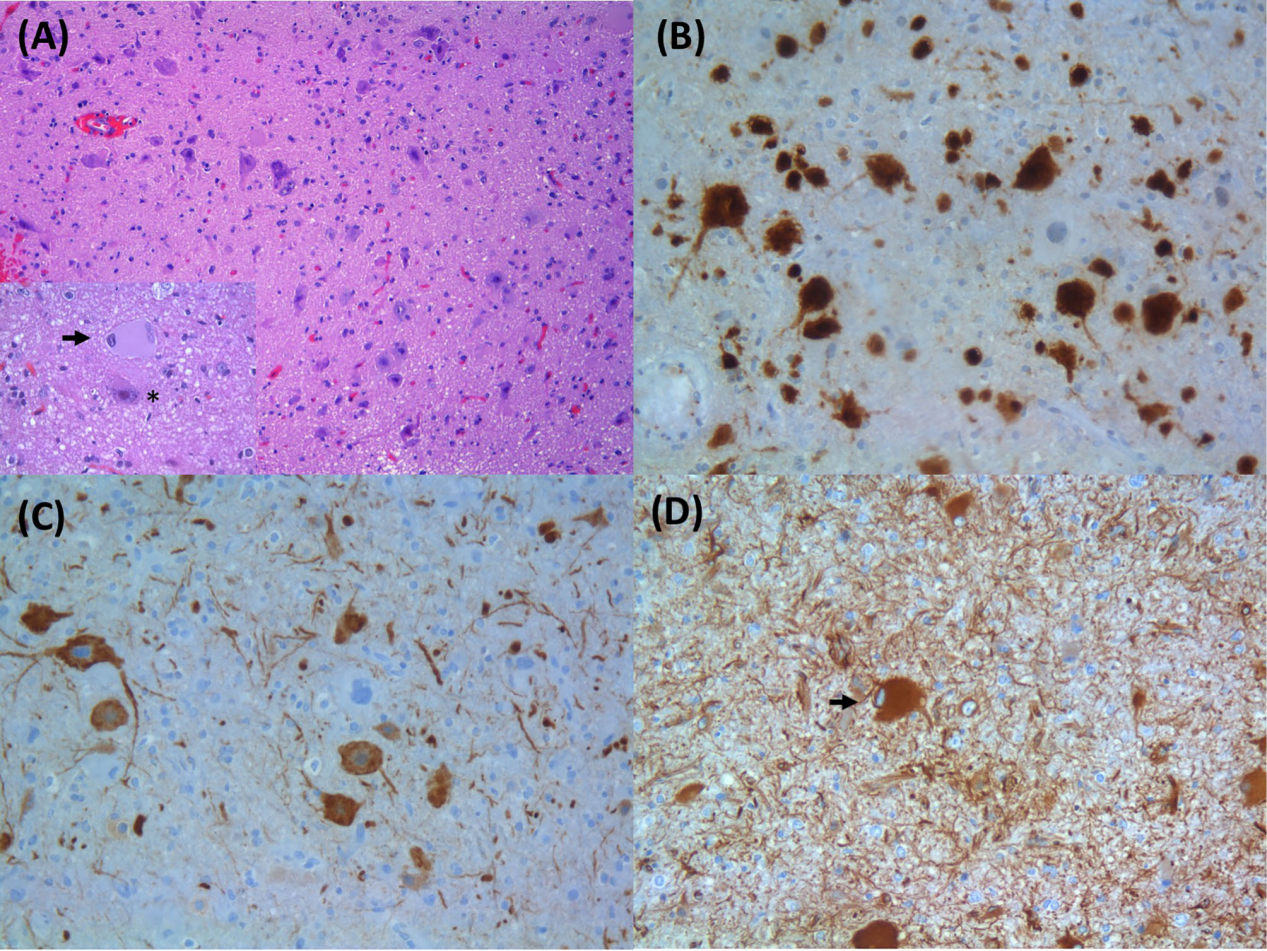
Histopathological findings of focal cortical dysplasia type IIB: **(A)** enlarged dysmorphic neurons and balloon cells; inset in **(A)** with a balloon cell (arrow) and dysmorphic neuron (asterisk) (HE, original magnification: 100× and 400×); **(B)** dysmorphic neurons expressing NeuN (IHC; original magnification: 200×); **(C)** dysmorphic neurons with aberrant accumulation of nonphosphorylated neurofilament proteins (SMI32 IHC; original magnification: 200×); **(D)** balloon cells express the intermediate filament vimentin (arrow) (IHC; original magnification: 200×). HE, hematoxylin and eosin; IHC, immunohistochemistry.

Epilepsy affects around 50 million people worldwide, making it one of the most common neurological diseases (Epilepsy, 2019). Focal cortical dysplasias (FCDs) are a heterogeneous group of localized malformations of cortical development that are commonly associated with drug-resistant epilepsy in both children and adults, often requiring resective neurosurgery. FCD was the histopathological diagnosis in approximately 20% of individuals who underwent epilepsy surgery (Lamberink et al., 2020). Malformations of cortical development (MCD) result from a disruption in different critical stages of human corticogenesis—progenitor proliferation, neuronal migration, and connectivity (Subramanian et al., 2019). The International League Against Epilepsy (ILAE) consensus classification, based on histopathological features, categorizes FCD in a three-level system: FCD type I characterized by abnormalities in cortical architecture, including radial (Ia), tangential (Ib), or both (Ic) cortical dyslamination; FCD type II presenting abnormal cortical lamination plus dysmorphic neurons (DNs), without balloon cells (BCs) (IIa) or with BCs (IIb) (Figure 1); and FCD type III when associated with other principal brain lesions (Blümcke et al., 2011). The ILAE classification was established in 2011, when the genetic basis of FCD was still unknown.

Over the past decade, insights in the etiology of FCD have been unraveled through next-generation sequencing (NGS) techniques. *De novo* brain-specific somatic mutations with low variant allelic frequencies were identified, a discovery only made possible using postsurgical dysplastic tissue. For FCD type II, most of the detected pathogenic variants converged in single genes regulating the mammalian target of rapamycin (mTOR) pathway, essential in neuronal growth and migration (Lim et al., 2015; Nakashima et al., 2015; Mirzaa et al., 2016; Moller et al., 2016). On the other hand, comprehensive descriptions on the genetic background of FCD type I are scarce. mTOR signaling cascade does not seem to be the principal target, unveiling that the broad spectrum of FCD may represent different molecular entities (Uddin et al., 2017; Baldassari et al., 2019b; Sim et al., 2019). mTOR is a serine/threonine protein kinase, member of the phosphoinositide 3-kinase-(PI3K)-related kinase family, that regulates fundamental cell physiological processes in response to different cellular inputs (e.g., growth factors, nutrients) (Marsan and Baulac, 2018; Jesus-Ribeiro et al., 2020). mTOR integrates two distinct protein complexes: mTORC1 when bound to the subunit Raptor (regulatory-associated protein of mTOR) and mTORC2 when associated to the subunit Rictor (rapamycin-insensitive companion of mTOR) (Marsan and Baulac, 2018; Jesus-Ribeiro et al., 2020). mTORC1 is the best characterized of the two mTOR complexes and is a central regulator of protein and lipid synthesis, ribosome biogenesis, cell growth and proliferation, metabolism, and autophagy (Marsan and Baulac, 2018; Jesus-Ribeiro et al., 2020). mTORC2 controls cell proliferation and survival and regulates actin cytoskeleton (Marsan and Baulac, 2018; Jesus-Ribeiro et al., 2020).

Emerging evidence is also starting to elucidate the functional role of epigenetic factors in the pathophysiology of FCD. Differential DNA methylation has been involved in the regulation of potential gene networks, like the mTOR pathway, in FCD type II (Dixit et al., 2018). Moreover, some FCD subtypes (Ia, IIa, and IIb) can be distinguished based on DNA methylation profiles, highlighting the possibility of disease-specific signatures to be used as biomarkers (Kobow et al., 2019). Dysregulation of microRNAs, a class of small non-coding single-stranded RNA molecules that act as posttranscriptional regulators of gene expression, may contribute to a failure in the neuronal differentiation and migration process (Wang et al., 2016; Che et al., 2017; Avansini et al., 2018).

To overcome the suboptimal long-term postsurgical outcome of FCD (Engel class I at 5 years: 54.5 and 67.4% for patients with FCD types I and II, respectively) (Lamberink et al., 2020), it is important to understand FCD pathogenesis, to develop an integrative clinicopathologic and molecular diagnosis, and to define specific target therapeutics. In order to contribute toward that progress, this review pools studies on genomic and epigenetic findings in postoperative human tissue of FCD types I and II to provide an updated overview on the molecular basis of these entities. We aimed to answer the following research questions:

1. What are the genes and biological pathways underpinning FCD etiology?
2. Is it possible to draw a genotype–histopathological phenotype correlation?
3. What are the epigenetic mechanisms involved in FCD pathogenesis and their biological relevance?

Also, the ultimate goal is to pinpoint knowledge gaps in the literature that can guide future translational research.

## Methods

### Study Design and Search Strategy

A scoping review of the literature was performed systematically according to previously described methods and the PRISMA-ScR Checklist (Arksey and O'Malley, 2005; Levac et al., 2010; Peters et al., 2015; Tricco et al., 2018). The search strategy was delineated with the assistance from a health sciences librarian with experience in search methodologies. A comprehensive literature search, divided in genomic and epigenetic fields, was conducted on PubMed, Embase, and Web of Science Core Collection, from inception to 07 May 2020. A combination of free text words relevant to this review and subject headings specific to each database was used. The search queries applied were included as Supplementary Table 1. Articles were retrieved from each database and imported into a reference management software. The references of included papers were also reviewed to identify any relevant papers that may have been missed in the initial search.

### Study Selection and Eligibility Criteria

In the first phase, records were screened for possible relevance by the lead authors (JJ-R, LMP), based on titles and abstracts. Subsequently, the relevant studies were assessed on their eligibility for inclusion dependent on full texts. Decisions about ambiguous papers were discussed together by two or more authors to reach a consensus. A paper was included for final data extraction when it met the following criteria: peer-reviewed original manuscript reporting novel gene variants or epigenetic regulatory mechanisms (e.g., DNA methylation, non-coding RNAs, histone posttranslational modifications) in patients that underwent epilepsy surgery, with clinico-imagiological and histopathological diagnosis of FCD type I or II according to Palmini's classification system proposed in 2004 (Palmini et al., 2004) or the ILAE classification system proposed in 2011 (Blümcke et al., 2011), published in English, Portuguese, Spanish, or Italian. Papers were excluded for the following reasons: irrelevant topics; no full-text available (conference abstracts); absence of a clear histopathological classification of the FCD subtypes; FCD type III or other coexisting brain pathologies, precluding any conclusion regarding to which lesion would be associated primarily with any molecular findings; other isolated MCD; exclusively *in vitro* or *in vivo* functional studies; no genetic findings of interest; and exclusively gene expression studies.

### Data Charting Process and Summary

Data extraction from the included published papers was performed independently by two authors through a qualitative content analysis approach, including the reference, study design, number of patients and controls, patients' clinical features (e.g., gender, age at surgery), type of samples used, neuropathological diagnosis, applied methodology, mutational description, and details on epigenetic mechanisms. The data were collated and summarized on a standardized evidence table.

## Results

**Figure 2:**
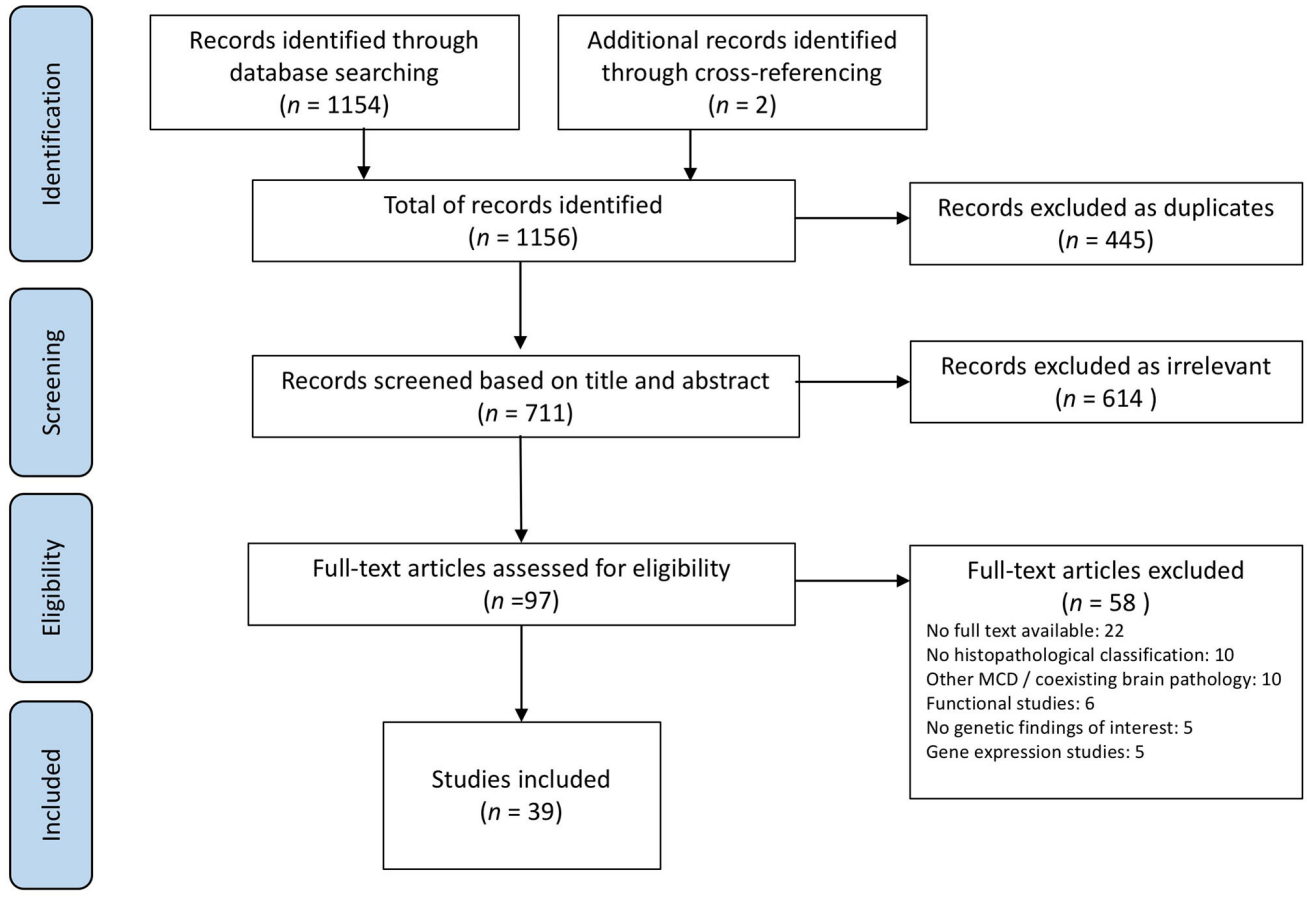
PRISMA flowchart of the study selection process.

The initial search yielded 1,154 studies plus 2 additional records identified through cross-referencing, 39 of which met the defined criteria and were included in the scoping review (Figure 2). Papers were published between 2002 and 2020 and comprised case reports and observational and experimental studies.

A synopsis of the results across studies was organized into two sections. First, we provide a descriptive summary of the genes and biological pathways affected in FCD types I and II (*n* = 33). Second, we synthesize the data from the epigenetic content of the papers including the different regulatory machinery (*n* = 8). Two papers were included in both sections since they covered both genetic and epigenetic fields. There were 5 studies with FCD type I, 19 studies with FCD type II, and 15 with both.

### Genetic Background of FCD

#### Patients' Characteristics and Study Design

A total of 648 patients were enrolled in the studies. All of them had medically refractory epilepsy amenable by surgical treatment, with resection of the epileptogenic zone after a complete preoperative evaluation. The studies included mainly children (84.3%), of which 51.6% were male, and the mean age at surgery was 7.1 ± 6.1 years (min. 0.1–max. 32). A section of the resected brain specimen was formalin-fixed and paraffin-embedded (FFPE) for the neuropathological classification in FCD type I, IIa, or IIb, and an adjacent block was immediately frozen in liquid nitrogen or stored at −80°C for research purposes (Lim et al., 2015; Nakashima et al., 2015; Baldassari et al., 2019b; Zhao et al., 2019; Lee et al., 2020; Zhang et al., 2020).

The experimental design employed by various studies comprised an initial deep whole exome sequencing using matched samples of peripheral blood lymphocytes or saliva and surgically resected fresh-frozen or formalin-fixed paraffin-embedded brain tissue, followed by bioinformatics analysis of the raw sequencing data and variant identification, with final validation using various targeted sequencing methods with high coverage (>100 ×) (Lim et al., 2015, 2017; Nakashima et al., 2015; Moller et al., 2016; Baldassari et al., 2019b; Sim et al., 2019; Zhao et al., 2019; Zhang et al., 2020). Subsequently, several studies performed *in vitro* functional analysis, with kinase assays and immunohistochemistry/immunoblotting for the detection of phosphorylated upstream (phospho-AKT) (Schick et al., 2006; Conti et al., 2015; Jansen et al., 2015; Mirzaa et al., 2016) or downstream (phospho-S6 ribosomal or 4EBP proteins) mediators of the mTOR signaling pathway in pathological brain samples and transfected heterologous mammalian cell lines (HEK293T) (Lim et al., 2015, 2017; Nakashima et al., 2015; Moller et al., 2016; Ribierre et al., 2018; Zhao et al., 2019; Zhang et al., 2020). These findings in human tissue led investigators to ascertain the pathogenicity of the detected brain mosaic variants in mouse models, through *in utero* electroporation combined, in some cases, with the CRISPR-Cas9 gene editing system (Lim et al., 2015, 2017; Ribierre et al., 2018; Zhao et al., 2019).

#### Gene Mutations and Affected Biological Pathways

An overview of the genetic findings in FCD types I and II is represented in Supplementary Table 2.

##### Focal Cortical Dysplasia Type I

Germline [*PCDH19* (Kurian et al., 2018), *SCN1A* (Barba et al., 2014), *KCNT1* (Rubboli et al., 2019), *STXBP1* (Weckhuysen et al., 2013; Uddin et al., 2017), *DEPDC5* (Baulac et al., 2015; Baldassari et al., 2019a), *NPRL2* (Weckhuysen et al., 2016)] and somatic [*STXBP1* (Uddin et al., 2017), *AKT3* (Conti et al., 2015), *DEPDC5* (Baulac et al., 2015), *SLC35A2* (Winawer et al., 2018)] variants were reported in 28.6% (14 out of 49) FCD I individuals, affecting a very diverse repertoire of genes. Pathogenic variants in the protocadherin 19 gene (*PCDH19*), located at Xq22, were originally reported in females with epilepsy and intellectual disability and, in a small percentage, Dravet-like syndrome phenotype (Kurian et al., 2018). It is expressed during brain development and is thought to be involved in neuronal migration and establishment of synaptic connections (Kurian et al., 2018). Ion channelopathies like α1-sodium channel subunit (*SCN1A*) and sodium-activated potassium channel gene (*KCNT1*), typically associated with Dravet syndrome and sleep-related hypermotor epilepsy, respectively, can co-occur with histologically proven FCD (Barba et al., 2014; Rubboli et al., 2019). Whether the coexistence of these two highly epileptogenic substrates is casual or causally related is not known. Germline variants and dysplasia-specific mosaicism of *STXBP1* were also implicated in FCD type I (Weckhuysen et al., 2013; Uddin et al., 2017). *STXBP1* encodes a syntaxin-binding protein that plays an important role in the release of neurotransmitters through the regulation of syntaxin (Uddin et al., 2017). Disruption of the mTOR pathway by mutations in related genes (*AKT3, DEPDC5, NPRL2*) has been reported as well (Baulac et al., 2015; Weckhuysen et al., 2016; Baldassari et al., 2019a). *SLC35A2* (locus Xp11.23) encodes a UDP-galactose transporter that permits the transport of galactose necessary for glycosylation (Winawer et al., 2018). Non-synonymous variants in *SLC35A2* were detected in resected FCD I tissue but not in the blood and were predicted to be deleterious. Importantly, phospho-S6 staining was not enhanced in the resected samples. Therefore, *SLC35A2* variants do not appear to be acting through activation of mTOR signaling (Winawer et al., 2018).

Several studies including FCD type I (35 patients in total) were unable to find somatic mutations in mTOR pathway-related genes (Jansen et al., 2015; Nakashima et al., 2015; Moller et al., 2016; Sim et al., 2016) and N-glycosylation pathway-associated *SLC35A2* gene (Baldassari et al., 2019b; Sim et al., 2019). Functional assessment of mTOR complex 1 (mTORC1) activity in FCD I tissue was also contradictory between studies. While some authors observed a hyperactivation of the PI3K/AKT/mTOR pathway (Conti et al., 2015; Weckhuysen et al., 2016), others reported no apparent phospho-S6 immunoreactivity in FCD I specimens (Nakashima et al., 2015; Winawer et al., 2018). Some studies highlighted another limitation in the classification of FCD since it cannot always be unequivocally diagnosed by histopathological assessment due to the sampling constraints (small size and fragmented samples) and the within-specimen variability which may not be representative (incomplete resection with a suspected macroscopic lesion in the vicinity) (Baulac et al., 2015; Weckhuysen et al., 2016; Winawer et al., 2018). Hence, in two studies involving mTOR-related genes (*DEPDC5* and *NPRL2*), although histology revealed an FCD type I, macroscopic anatomy or clinical and imagiological findings suggested an FCD type II (Baulac et al., 2015; Weckhuysen et al., 2016).

Consistent with this, the available data preclude a reliable conclusion regarding the underlying pathogenetic mechanism in FCD type I.

##### Focal Cortical Dysplasia Type II

**Figure 3:**
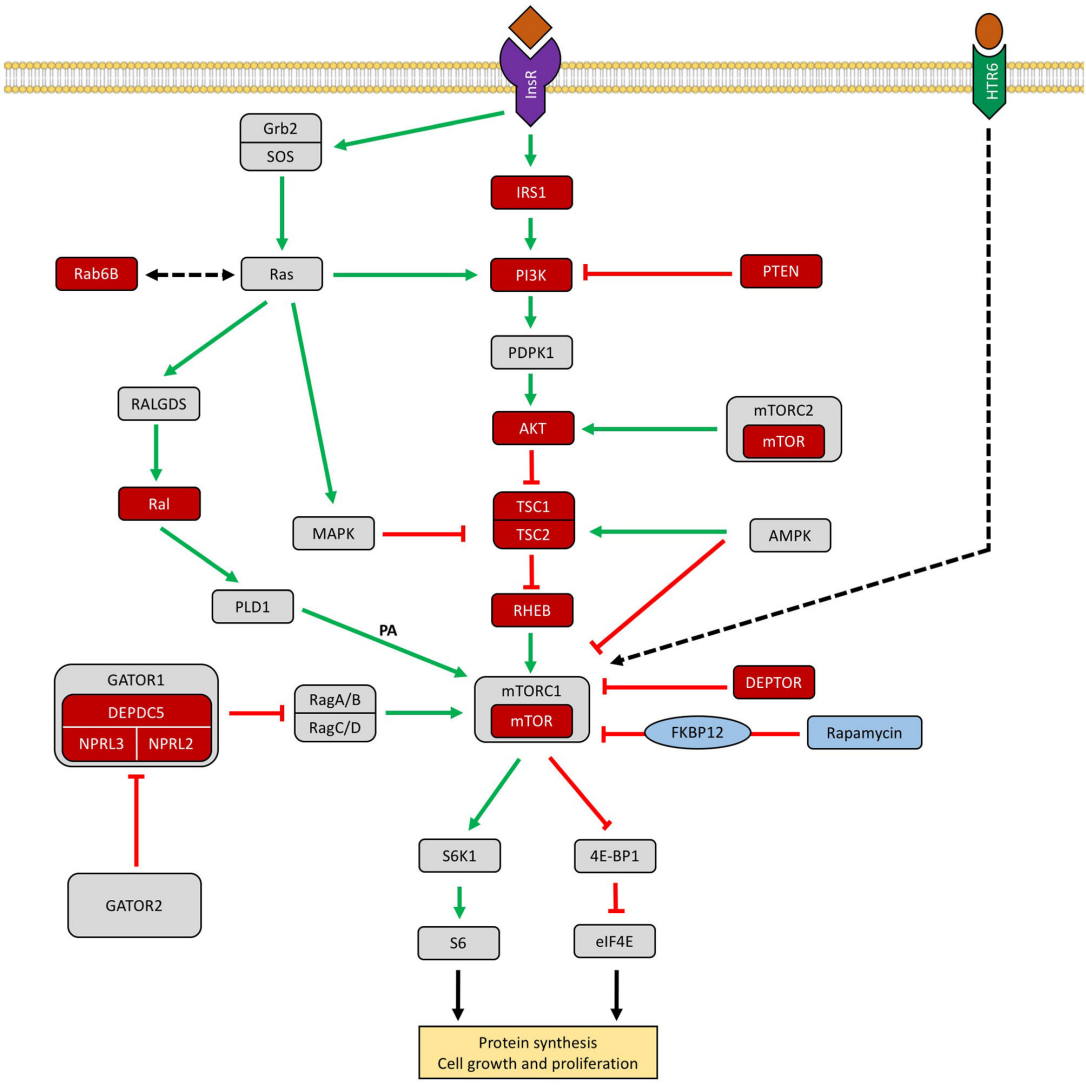
Schematic representation of the PI3K–AKT–mTOR signaling cascade and other related pathways involved in FCD. The GATOR1 (Gap Activity Toward Rags 1) complex inhibits mTORC1 in amino acid deprivation conditions. Genes encoding proteins reported in patients with focal cortical dysplasias are colored in red. Rapamycin (sirolimus) is an effective inhibitor of mTORC1 *via* FKBP12 (Iffland and Crino, 2017; Marsan and Baulac, 2018; Jesus-Ribeiro et al., 2020; Zhang et al., 2020). InsR, insulin receptor; IRS1, insulin receptor substrate 1; PTEN, phosphatase and tensin homolog protein; AKT, protein kinase B; TSC1/2, tuberous sclerosis complex subunit 1/2; Rheb, GTPase Ras homolog enriched in brain; DEPDC5, DEP domain containing 5; NPRL 2/3, nitrogen permease regulator-like 2 and 3; DEPTOR, DEP domain-containing mTOR-interacting protein; AMPK, AMP-activated protein kinase; MAPK, mitogen-activated protein kinase pathway; S6K1, p70 ribosomal protein S6 kinase ½; 4E-BP1, eukaryotic translation initiation factor 4E binding protein 1; RAB6B, Ras-related protein Rab-6B, Ral, Ras-like proto-oncogene, HTR6, 5-hydroxytryptamine receptor 6, FKBP12, FK506-binding protein-12.

A schematic representation of the PI3K–AKT–mTOR signaling cascade and other related pathways involved in FCD pathogenesis can be seen in Figure 3.

*MTOR Gene and the mTOR Complex 1.* Brain-only somatic *MTOR* mutations accounted for 3.8–57.1% of patients with FCD II across different studies and represented the most commonly affected gene (Leventer et al., 2015; Lim et al., 2015; Nakashima et al., 2015; Mirzaa et al., 2016; Moller et al., 2016; D'Gama et al., 2017; Avansini et al., 2018; Baldassari et al., 2019b; Sim et al., 2019; Zhang et al., 2020). The variant allelic frequencies (VAF) detected in the brain-derived DNA were low, ranging from 0.25 to 15.67% for FCD IIa and from 0.74 to 10.6% for FCD IIb (Leventer et al., 2015; Lim et al., 2015; Nakashima et al., 2015; Mirzaa et al., 2016; Moller et al., 2016; D'Gama et al., 2017; Avansini et al., 2018; Baldassari et al., 2019b; Sim et al., 2019; Zhang et al., 2020). All *MTOR* somatic variants were missense, leading to a substitution of an amino acid in the protein. Recurrence of p.Ser2215Phe (nine times), p.Leu1460Pro (eight times), p.Ser2215Tyr (seven times), p.Thr1977Lys (four times), p.Ala1459Asp (four times), and p.Cys1483Arg (three times) mutations in several studies, using distinct technologies and sequencing platforms, highlighted mutational hotspot clustering in FAT and kinase domains of the mTOR protein (Moller et al., 2016; Baldassari et al., 2019b).

The identified somatic *MTOR* mutations caused aberrant activation of mTORC1 in *in vitro* functional assays (Lim et al., 2015; Nakashima et al., 2015; Mirzaa et al., 2016; Moller et al., 2016), suggesting a gain-of-function as the pathogenetic mechanism. Distinct variants (p.Ser2215Phe, p.Thr1977Lys) affecting the *MTOR* gene do not seem to confer different levels of mTOR activation based on pS6-immunostaining profile (Baldassari et al., 2019b). Focal cortical expression of mutant *MTOR* (p.Leu2427Pro) by *in utero* electroporation in mice was sufficient to induce neuronal migration defect and DNs, as well as spontaneous seizures (Lim et al., 2015). Treatment with rapamycin, a clinically approved mTORC1 inhibitor drug, reversed the cytological abnormalities and the seizures (Lim et al., 2015).

No pathogenic variants were reported affecting the specific subunits of mTORC1 (*RPTOR*) and mTORC2 (*RICTOR*) (Baldassari et al., 2019b; Kobow et al., 2019).

*The PI3K–PTEN–AKT–TSC–RHEB Pathway.* Individuals negative for *MTOR* mutations can harbor somatic mutations in genes encoding upstream mediators of the mTOR signaling cascade. Brain mosaic activating mutations in *PIK3CA*, with VAF of 4.7%, have been reported in FCD IIa (Jansen et al., 2015). Missense germline variants of uncertain significance in *PIK3C3* and *PIK3C2B* were observed in two cases of FCD IIb (D'Gama et al., 2015). Cell-type-specific analysis using laser microdissection of DNs and BCs detected a *PTEN* somatic missense variant, not present in the adjacent normal brain tissue of the same patient, and associated with substantial expression of phospho-Akt (Schick et al., 2006). Variants of unknown significance in the *AKT1* gene were detected in FCD type IIa and IIb tissues (Avansini et al., 2018; Kobow et al., 2019), Baldassari et al. (2019b) reported two patients with FCD IIa with a somatic missense mutation (p.Glu17Lys) in *AKT3*, with VAF of 1.1–2.3%.

Loss-of-function mosaic mutations in negative regulators of mTOR kinase, as *TSC1* and *TSC2*, induce mTOR hyperactivation by disturbing the function of the TSC1–TSC2 complex (Lim et al., 2017; Baldassari et al., 2019b). The missense, non-sense, and frameshift indel variants detected in *TSC1* and *TSC2* genes were present in both FCD type IIa and IIb specimens, with VAF ranging from 1.0 to 6.7% (D'Gama et al., 2017; Lim et al., 2017; Avansini et al., 2018; Baldassari et al., 2019b; Sim et al., 2019; Zhang et al., 2020). Contrary to the germline variants, somatic *TSC* mutations were associated with isolated FCD without the typical systemic tuberous sclerosis complex clinical manifestations (D'Gama et al., 2017; Lim et al., 2017; Baldassari et al., 2019b). *In utero* CRISPR-Cas9-mediated genome editing of *Tsc1* and *Tsc2* in a small fraction of neurons in the embryonic mouse brain recapitulated the clinical and histopathological phenotype of FCD II, which was reversed with rapamycin treatment.

A brain somatic doublet mutation in FCD IIa (Zhao et al., 2019) and a somatic mutation in FCD IIb (Baldassari et al., 2019b) have been observed targeting the *RHEB* gene, with VAF of 5.8–8.8%. *RHEB* functions as a crucial connection between the PI3K–AKT–TSC and mTOR pathways. RHEB p.Tyr35Leu mutant expression was associated with overactive mTORC1 signaling, as evidenced by elevated phosphorylated S6 (Zhao et al., 2019). Also, embryonic expression of the RHEB p.Tyr35Leu led to FCD II-like phenotype in mice, which was attenuated by treatment with rapamycin (Zhao et al., 2019).

*GATOR1 Complex Encoding Genes.* DEPDC5, a GTPase-activating protein, together with NPRL2 and NPRL3 forms the GATOR1 complex, an upstream repressor of mTORC1. Germline *DEPDC5* mutations were the second most common genetic scenario in FCD II, mainly in FCD IIa cases (77.8%) (Baulac et al., 2015; Carvill et al., 2015; D'Gama et al., 2015, 2017; Scerri et al., 2015; Baldassari et al., 2019a; Kobow et al., 2019; Ying et al., 2019). Variants were often dominantly inherited from an asymptomatic carrier parent, illustrating the incomplete penetrance of *DEPDC5* variants (Baulac et al., 2015; Sim et al., 2016; Baldassari et al., 2019a). Among the distinct variants reported, null variants (non-sense and frameshift) resulting in a premature stop codon were the most frequent type (65.7%, 23 of 35). A possible explanation on how a germline variant affecting a ubiquitous pathway may cause a focal dysplastic malformation is the presence of a somatic second hit, supporting the application of Knudson's two-hit model of tumor pathogenesis (Ribierre et al., 2018; Baldassari et al., 2019b; Sim et al., 2019; Lee et al., 2020). This biallelic gene inactivation mechanism of *DEPDC5* in a subset of brain cells was confirmed by two different studies, which provided compelling evidence for a heterozygous germline and a second allele somatic hit in *trans* configuration in FCD IIa (Ribierre et al., 2018; Lee et al., 2020). In addition, a somatic second-hit loss-of-heterozygosity in a *DEPDC5* germline case was unveiled by single-cell microdissection followed by sequencing of enriched pools of DNs (Baldassari et al., 2019b). Increased phosphorylation levels of the protein S6 were demonstrated in DNs present in the resected brain specimens, implying a mTORC1 signaling hyperactivation (Scerri et al., 2015; Ribierre et al., 2018; Baldassari et al., 2019b; Ying et al., 2019). Depdc5 brain mosaic inactivation using CRISPR-Cas9 editing and *in utero* electroporation in a mouse model induced the aberrant neuropathological hallmarks of FCD II, which were rescued by the treatment with rapamycin (Ribierre et al., 2018).

Variants in *NPRL2* (D'Gama et al., 2017; Baldassari et al., 2019a) and *NPRL3* (Sim et al., 2016; Weckhuysen et al., 2016) were described merely in FCD subtype IIa. *NPRL2* and *NPRL3* mutations caused aberrant hyperactivation of mTORC1 (Weckhuysen et al., 2016). Haploinsufficiency is the pathogenic mechanism in GATOR1-related disorders (Carvill et al., 2015; Sim et al., 2016; Weckhuysen et al., 2016; Baldassari et al., 2019a). *NPRL3* variants led to nonsense-mediated decay of the mutant messenger RNA in cultured lymphoblastoid cells (Weckhuysen et al., 2016) and in FCD IIa resected tissue (Sim et al., 2016), suggesting a loss-of-function mechanism. More studies are needed to explore the presence of a second-hit somatic mutation in the negative regulators *NPRL2* or *NPRL3*.

No pathogenic variants have been found in GATOR2 complex genes (*MIOS, SEC13, SEH1L, WDR24*, and *WDR59*) (Weckhuysen et al., 2016; Baldassari et al., 2019b).

*Other Reported Genetic Variants.* Although multiple studies have demonstrated that pathogenic variants in mTOR pathway-related genes are one of main genetic causes of FCD type II, in up to approximately half of the patients, it was not possible to detect a putative mutation. Other potentially deleterious somatic variants in five novel genes, not previously associated with cortical malformations, have been identified: insulin receptor substrate 1 (*IRS1*), Ras-related protein Rab-6B (*RAB6B*), zinc finger protein 337 (*ZNF337*), Ras-like proto-oncogene A (*RALA*), and 5-hydroxytryptamine receptor 6 (*HTR6*) (Zhang et al., 2020). Through a pathway analysis, four of these genes (*IRS1, RAB6B, RALA*, and *HTR6*) interacted with the mTOR signaling cascade, highlighting the probable pleomorphism of FCD pathogenesis with involvement of other circuits like insulin and Ras pathways. These pathways are not specific for FCD, with documented involvement in other pathologies like cancer and neurofibromatosis type 1 (Zhang et al., 2020). *HTR6* is a protein-coding gene for the receptor of 5-hydroxytryptamine, a biogenic hormone that functions as a neurotransmitter important for brain development (Zhang et al., 2020). An *in vitro* functional study demonstrated that the *IRS1* variant led to mTORC1 hyperactivation in both HEK293T transfected cells and in dysmorphic neurons and balloon cells in FCD II lesions (Zhang et al., 2020). The role in the pathogenesis of FCD II of the potentially deleterious somatic variants in the brain-expressed genes *RAB6B, ZNF337, RALA*, and *HTR6* was not determined.

Rare patients with germline genetic syndromes, affecting *SCN1A* (Barba et al., 2014) and *PCDH19* (Kurian et al., 2018), have been described as having FCD type II on histopathological assessment. However, the limited number of patients with epilepsy surgery, the broad range of MCD described (different FCD subtypes and periventricular nodular heterotopia), and the absence of DNA sequencing from postoperative brain tissue make it difficult to confirm a disease-causing function of these variants in FCD development. Still, the hypothesis of a second-hit somatic mutation model that would support the focal cortical lesion similar to the one described for *DEPDC5* cannot be refuted and additional studies are needed.

##### Genotype–Phenotype Correlation

A controversial point of discussion is whether FCD type I shares the same genetic basis as FCD type II. An overview of the vast amount of published data reveals that probably FCD types I and II differ in its molecular background. Somatic gain-of-function variants in *MTOR* and its activators (*PIK3CA, AKT3, RHEB*) (Jansen et al., 2015; Lim et al., 2015; Nakashima et al., 2015; Mirzaa et al., 2016; Moller et al., 2016; Zhao et al., 2019), as well as germline, somatic, and second-hit mosaic loss-of-function variants in its related repressors (*PTEN, TSC1, TSC2, DEPDC5, NPRL2, NPRL3*)(Schick et al., 2006; Baulac et al., 2015; Sim et al., 2016; Weckhuysen et al., 2016; Lim et al., 2017; Ribierre et al., 2018; Baldassari et al., 2019b; Lee et al., 2020), have been reported as the major causes in FCD type II cases (D'Gama et al., 2015, 2017; Baldassari et al., 2019b; Sim et al., 2019). On the other hand, for FCD type I, the number of samples included in the studies is lower and the documented brain mosaic variants targeted a very pleomorphic spectrum of genes and respective biological pathways [*STXBP1* (Uddin et al., 2017), *AKT3* (Conti et al., 2015), *DEPDC5* (Baulac et al., 2015), *SLC35A2* (Winawer et al., 2018)]. Thus, although FCD type II can be classified mostly as an mTORopathy, any conclusion regarding FCD type I would need further investigation with larger patient series and a comprehensive approach integrating molecular–genetic investigations with a reliable histological phenotype.

Another striking question is whether FCD IIa and IIb subtypes represent distinct entities since they can be distinguished based on the presence or absence of BCs in histopathological evaluation (Najm et al., 2018). Brain somatic mTOR pathogenic variants were found indistinctly in both FCD IIa and FCD IIb. Genetic analysis demonstrated that the same variants in *TSC1* [c.64C>T (p.Arg22Trp)] and in *MTOR* [c.4376C>A (p.Ala1459Asp), c.4379 T>C (p.Leu1460Pro), c.6644C>T (p.Ser2215Phe), c.6644C>A (p.Ser2215Tyr), c.5930C>A (p.Thr1977Lys)] genes, with similar degrees of brain mosaicism, gave rise to both FCD phenotypes (IIa and IIb), suggesting that the two subtypes represent a gradient of the same disorder, therefore challenging the current classification (Lim et al., 2017; Baldassari et al., 2019b). These data were confirmed with single-cell microdissection followed by sequencing of enriched pools of pathological cells. Both enlarged DNs and BCs were the main carriers of the somatic pathogenic variants in FCD II cases, indicating that they most likely derive from the same cellular lineage (Schick et al., 2006; Baldassari et al., 2019b; Lee et al., 2020). However, the origin of BCs, found exclusively in FCD IIb, remains enigmatic.

The same mutated gene may present phenotype variability. It is related to the embryonic stage in which the postzygotic mutational event occurs and, consequently, to the fraction of brain cells carrying it. FCD II described as large or hemispheric had higher VAF in brain tissue, strongly indicating a relationship between the brain mosaic rate and the extent of the brain malformations (Leventer et al., 2015; D'Gama et al., 2017; Baldassari et al., 2019b). Therefore, extensive cortical dysplasias could be due to the occurrence of somatic mutations earlier during brain development. A mutation gradient with an epicenter in the seizure-onset zone, compared with the surrounding epileptogenic zone with lower mosaic rates, was observed in FCD type II tissue (Mirzaa et al., 2016; Ribierre et al., 2018; Baldassari et al., 2019b; Lee et al., 2020). Somatic mutation load correlated with the density of pathological cells (DNs or BCs) (Baldassari et al., 2019b; Lee et al., 2020).

The complexity of FCD genetics has also been brought to light since a few studies have shown that one patient can harbor more than one variant in two different genes (Avansini et al., 2018; Zhang et al., 2020), proposing that somatic variants can co-occur and accumulate during the embryonic cell proliferation process (Zhang et al., 2020). Accordingly, it is not possible to exclude the hypothesis of a digenic/oligogenic etiology of FCD and more studies with single-cell sequencing are necessary.

### Epigenetic Regulatory Mechanisms in FCD

Epigenetics represents heritable modifications in gene expression that do not result from an alteration in the DNA sequence (Kobow and Blümcke, 2018). The different epigenetic processes can act either in a synergistic or in an antagonistic way, showing variability and interdependence (Kobow and Blümcke, 2018). DNA methylation and microRNAs were the two epigenetic mechanisms documented in both FCD types I and II. The overall epigenetic findings are presented in Supplementary Table 3.

#### DNA Methylation

Two studies analyzing differentially methylated genes through distinct methodologies, namely genome-wide DNA methylation microarrays (Dixit et al., 2018) and sequencing (Kobow et al., 2019), showed the biological relevance of methylation as a regulatory mechanism in FCD.

Integrative analysis of DNA methylation and RNA sequencing data in FCD type II postoperative tissue revealed an inverse correlation between promoter methylation and gene expression (Dixit et al., 2018). Some of these genes were evolved in RTK (*EGFR, PDGFRA, NTRK3*) and mTOR (*RPS6KA3, PRKAA1*) signaling pathways, as well as synaptic transmission (*KCNH8, DLG1*), neuronal development, and cell–cell interaction networks (*NEUROD1, NR4A3, ECT2, BCL6, NF-kB2, BRCA1, UNC5B*) (Dixit et al., 2018). In mTOR pathway-related genes, *RPS6KA3*, the ribosomal protein S6 kinase A3 which phosphorylates members of the MAPK pathway and TSC2, was upregulated, and *PRKAA1*, the catalytic subunit of AMPK, was downregulated, both resulting in increased mTORC1 signaling (Dixit et al., 2018). Upregulation of DNMT3α suggested an active process of *de novo* methylation (Dixit et al., 2018).

Differential DNA methylation was able to discriminate between different subtypes of FCD (Ia, IIa, and IIb), other epilepsy phenotype (temporal lobe epilepsy), and non-epilepsy age-matched controls (Kobow et al., 2019). However, gene expression was less reliable for FCD classification (Kobow et al., 2019). Particularly, for mTOR pathway-related genes, no enrichment of differential methylation or gene expression was identified (Kobow et al., 2019).

As opposed to brain tumors, that seem to have promoter-centered DNA methylation changes, in both studies with FCD tissue, promoters accounted only for 12 and 35% of the differential methylation sites, respectively, with higher percentages for gene bodies (Dixit et al., 2018; Kobow et al., 2019).

#### MicroRNAs

Recent research has identified a possible role for microRNAs in the regulation of target genes in FCD. Several microRNAs, like miR-17~92 cluster, hsa-miR-21, hsa-miR-155, hsa-miR-4521, miR-323a-5p, hsa-let-7f, has-miR-31, and hsa-miR-34a, have been reported to be differentially expressed in FCD type I and/or II compared with normal cortical tissue (Dogini et al., 2012; Lee et al., 2014; Wang et al., 2016; Che et al., 2017; Avansini et al., 2018).

miR-323a-5p and hsa-miR-4521 were upregulated in both the dysplastic cortex and plasma/serum of FCD patients, suggesting that these endogenous RNA molecules can function as a potential diagnostic biomarker for refractory epilepsy associated to FCD (Wang et al., 2016; Che et al., 2017). The plasma level of miR-323a-5p expression was positively correlated with both duration of epilepsy and seizure frequency, and it was significantly higher in patients with Engel class III/IV compared with Engel class I/II, implying that high miR-323a-5p expression may be correlated with poor prognosis in patients with FCD (Che et al., 2017). Still, a crucial question was whether differential expression of microRNAs was an initial event, suggesting a possible involvement in the development of FCD, or a secondary event related to seizure recurrence in chronic drug-resistant epilepsy, regardless of the etiology. Indeed, a hypothetical molecular mechanism in which microRNA dysregulation may be detrimental to early neuronal differentiation and migration was reported. Three microRNAs (hsa-let-7f, has-miR-31, and hsa-miR-34a) were downregulated in FCD type II tissue, compared with normal cortical tissue from autopsy and patients with mesial temporal sclerosis (Avansini et al., 2018). In addition, an overexpression of *NEUROG2*, a member of the family of transcription factors bHLH (basic helix loop-helix) involved in mammalian neurogenesis, and its target *RND2* was demonstrated. *NEUROG2* expression was regulated by hsa-miR-34a interaction with its 5′-UTR region, suggesting that the observed reduction in hsa-miR-34a expression may lead to a less efficient repression of *NEUROG2* (Avansini et al., 2018). *In situ* hybridization localized *NEUROG2* expression in BCs as well as in DNs (Avansini et al., 2018). Disruption in the interaction between miR-34a and *NEUROG2* led to an upregulation in the expression of *NEUROG2* and *RND2*, affecting the inhibition of neurogenesis and leading to an abnormal neuronal migration and differentiation, with the formation of aberrant cells (Avansini et al., 2018).

Other putative target genes of the differentially expressed microRNAs belonged to the mTOR pathway and LIS1 pathway, both involved in neuronal migration and cortical formation, and the Hippo signaling pathway, involved in the regulation of cell proliferation and organ size during development (Lee et al., 2014; Li et al., 2016). A cross talk between the Hippo pathway and the mTOR pathway may play a crucial role in the pathogenesis of cortical malformations (Li et al., 2016).

Similar to DNA methylation profiles, microRNAs were differentially expressed between FCD subtypes, suggesting that distinct forms of FCD had different molecular signatures (Dogini et al., 2012; Lee et al., 2014). The heterogeneity of the patients in terms of pathological subtype, lesion location, and age is an important limitation of the microRNA expression profiling studies (Li et al., 2016).

## Discussion

This scoping review summarizes existing evidence on genetic and epigenetic background of FCD types I and II, allowing a broader analysis of the results and limitations of the various studies and highlighting future research perspectives. The number of publications has risen significantly in the past decade, with only two articles prior to 2010 included. The dramatic increase in published papers likely reflects the advent of NGS and the availability of pathological brain specimens from epilepsy surgery.

A milestone in FCD research was the identification of low-level postzygotic brain-specific mutations disrupting the mTOR pathway and creating a mosaic with a small proportion of variant-carrying cells intermixed with variant-negative cells in postsurgical tissue (Jansen et al., 2015; Lim et al., 2015, 2017; Nakashima et al., 2015; Mirzaa et al., 2016; Moller et al., 2016; D'Gama et al., 2017; Baldassari et al., 2019b). Single-cell sequencing in mutation-positive samples showed that somatic mutations were always present in the neuronal lineage but variably present in glia, suggesting that overactivation of the mTOR pathway in neurons is necessary and sufficient for the abnormal cortical development (D'Gama et al., 2017). Subsequently, conditional *PIK3CA* activation in the mouse cortex showed that abnormal mTOR activation in the excitatory neurons and glia, but not the interneuron lineage, induced cortical lamination defects and overgrowth (D'Gama et al., 2017). These data imply somatic mutations activating the mTOR pathway in dorsal telencephalic progenitors as a cause for cortical dysplasias (D'Gama et al., 2017). The resulting phenotype would be dependent on the affected cell-type progenitors and the timing of the mutational event during corticogenesis, with mutations occurring later generating smaller focal malformations (Marsan and Baulac, 2018). Based on this scenario, FCD II, cortical tubers (tuberous sclerosis complex), and hemimegalencephaly (HME), which share neuropathological features such as cortical dyslamination, DNs, and BCs, represent a continuum of the same mTOR-associated neurodevelopmental disorder, with higher brain mosaic rates in HME compared with FCD II (D'Gama et al., 2015; Jansen et al., 2015; Mirzaa et al., 2016; Marsan and Baulac, 2018; Baldassari et al., 2019b; Sim et al., 2019).

While the exact genetic basis of FCD type I remains elusive, for FCD type II, multiple somatic, germline, and second-hit mosaic variants in the mTOR pathway-related genes have been identified. The majority of the studies reported a non-synonymous single nucleotide variant in a specific gene. *MTOR* and *DEPDC5* were the most frequently affected genes in FCD type II, with different mutational mechanisms. Single somatic activating mutations in *MTOR* (Leventer et al., 2015; Lim et al., 2015; Nakashima et al., 2015; Mirzaa et al., 2016; Moller et al., 2016) and germline and second-hit mosaic mutations in *DEPDC5* (Baulac et al., 2015; Carvill et al., 2015; Scerri et al., 2015; Ribierre et al., 2018; Lee et al., 2020) led to excessive mTOR signaling. The *in utero* electroporation technique offered a practical way of modeling the effects of somatic mutations in the fetal cortex of mouse models, confirming the causative role of the identified variants. Embryonic expression of *TSC1/TSC2* (Lim et al., 2017), *RHEB* (Zhao et al., 2019), *MTOR* (Lim et al., 2015), and *DEPDC5* (Ribierre et al., 2018) variants in a small fraction of neurons induced FCD II-like phenotypes in mice.

Nevertheless, a significant proportion of patients remain with negative sequencing results. How can we explain these missing mutational causes? Mutations can be present in not yet recognized genes, as highlighted by Zhang et al. who identified new candidate genes (*IRS1, RAB6B, ZNF337, RALA, HTR6*) (Zhang et al., 2020), which may or may not influence the mTOR pathway. Also, these mutations can affect non-coding regions of the genome, which might be unveiled in the future by deep whole-genome sequencing (Sim et al., 2019). Another alternative explanation is that a low density of pathological variant-carrying cells (DNs and BCs) in the tissue sample may drive to brain mosaicism below the threshold level of detection (Baldassari et al., 2019b). The higher the density of DNs and BCs, the greater the mutation load will be and, consequently, the variant detection likelihood (Baldassari et al., 2019b; Lee et al., 2020). Single-cell sequencing or enrichment of pathological cells by laser microdissection can overcome this obstacle (Sim et al., 2019). Additionally, the depth of sequencing is crucial to achieve optimal diagnostic yield, since somatic mutations with a mutational burden as low as 1% have been reported in the affected brain tissue (Marsan and Baulac, 2018; Baldassari et al., 2019b; Sim et al., 2019). VAF of less than 5%, which are unlikely to be detected with conventional NGS and Sanger sequencing, were reported in 77.8% (84 of 108) of the FCD II cases. High read depth sequencing, at least 1,000 ×, is required to confidently detect low allele frequency variants (Baldassari et al., 2019b; Sim et al., 2019). However, sequencing at high coverage may lead to a substantial amount of artifactual somatic mutations with low VAF, which may significantly lower positive predictive values (PPV) (Sim et al., 2019). The preservation method of the resected brain tissue, such as FFPE tissue specimens which are the most frequently available samples in clinical practice, can also negatively affect the detection accuracy of low-level somatic mutations (Sim et al., 2019). Sim et al. demonstrated that brain-only unmatched FFPE samples had the lowest PPV (11.1%), indicating that these samples are likely to produce more false-positive calls, which can be removed by an orthogonal validation method (site-specific amplicon sequencing or digital droplet PCR) (Sim et al., 2019). Importantly, this ever-increasing catalog of genetic findings was accompanied by new challenges in sequence interpretation, particularly for missense variants (Richards et al., 2015). The reported mosaic variants were detected across multiple studies using distinct technologies and sequencing platforms, and the assignment of pathogenicity was supported by *in vitro* and *in vivo* functional assays, which are considered strong evidence for pathogenicity according to the ACMG guidelines (Richards et al., 2015). Finally, epigenetic machinery may modulate gene expression and be accountable for some mutation-negative patients.

The dynamics of epigenetic processes, responsible for the regulation of normal brain activity, are gaining attention as possible dysregulated systems among different epilepsy etiologies (Hauser et al., 2018). DNA methylation and noncoding RNAs, such as microRNAs, are the two mechanisms implicated in the pathogenesis of FCD and influence phenotypic manifestations (Dogini et al., 2012; Lee et al., 2014; Li et al., 2016; Wang et al., 2016; Che et al., 2017; Avansini et al., 2018; Dixit et al., 2018; Kobow et al., 2019). These epigenetic mechanisms may function as molecular signatures for FCD subtypes, representing potential clinically useful biomarkers and drug targets (Dogini et al., 2012; Lee et al., 2014; Kobow et al., 2019). However, further studies are needed to confirm that these epigenetic patterns are specific for FCD and not shared with other entities in the MCD group. A new research perspective may come with single-cell genomics. Since both DNs and BCs are variant-carrying cells, a comprehensive profiling of cell-type-specific methylome for these aberrant cells may explain the different histopathological phenotypes (IIa vs. IIb) and their individual contribution to the epileptic network.

Taken together, these data indicate that incorporating the genetic and epigenetic findings into neuropathological diagnosis, similar to the current recommended practice for brain tumor diagnostic work-up [WHO classification (Louis et al., 2016)], will help to refine diagnostic categorization and subsequently optimize treatment. A two-tiered approach into an integrated pathological diagnosis in epilepsy surgery has been proposed by Benova and Jacques (2019). Future progress will build on such comprehensive analysis to develop a targeted drug treatment, especially when epilepsy surgery is not an option or when the patient is not seizure-free after surgery. Currently, epilepsy surgery is a valuable option for drug-resistant epilepsy in mutation-positive patients with FCD (Baulac et al., 2015; Nakashima et al., 2015; Moller et al., 2016; Baldassari et al., 2019a, b). There is no predictive correlation between a given mutated gene and surgery outcome (Baldassari et al., 2019b). Mounting evidence supports the involvement of mTOR pathway genes in FCD II, raising the possibility that mTOR modulation might be a treatment option. *In vivo* functional studies revealed that rapamycin treatment suppressed the neuronal migratory defects and seizures in mouse models expressing the mTOR pathway-related variants found in FCD II tissue (Lim et al., 2015, 2017; Ribierre et al., 2018; Zhao et al., 2019). mTOR inhibitors, namely rapamycin and everolimus, which act by forming a complex with FK506-binding protein-12 (FKBP12) that inhibits mTORC1, have shown clinical benefit in tuberous sclerosis patients (Jesus-Ribeiro et al., 2020). A prospective, randomized, double-blind, placebo-controlled clinical trial (NCT03198949) is underway to evaluate the efficacy and safety of everolimus given as adjunctive therapy in patients with FCD type II for whom more than two antiepileptic drugs and surgery failed to control seizures. For *SLC35A2*-related patients, reported in FCD type I, treatment with exogenous galactose may be an interesting possibility, as observed previously in two cases (Dörre et al., 2015; Demos et al., 2019). Drugs targeting certain classes of epigenetic enzymes, like DNA methyltransferase (DNMT) inhibitors (e.g., nucleoside analogs—azacytidine, decitabine), are already used in the oncological field with success (Kobow and Blümcke, 2018). Despite that, novel DNA methyltransferase inhibitors with applicability in FCD and epilepsy are not yet a reality. Mouse models may serve as a valuable functional platform for future clinical research directed toward the development of novel brain-specific targeted therapeutics (Ribierre et al., 2018). It is tempting to speculate that genome and epigenetic editing technologies may foster further therapeutic breakthroughs for this pathology in the near future (Zhang et al., 2018).

In conclusion, throughout this paper, we focused on what we know regarding FCD genetic basis and what is still missing to point future directions in research. The pathogenetic mechanism behind FCD I, the expansion of the somatic second-hit model to other candidate genes, the first insights of epigenetic modifications in FCD, and new targeted therapeutics are just some of the challenging areas that need deeper investigation. Moreover, MCD animal models may offer an elegant way of studying the transduction of signal linking the mTOR signaling cascade hyperactivation to neuronal hyperexcitability, ultimately leading to epilepsy (Moller et al., 2016; Marsan and Baulac, 2018). However, there is no consensus about which model represents the ideal choice and what is the place for alternative models (organoids, induced pluripotent stem cells, transdifferentiated neuronal cells) (Sapir et al., 2019). Also, the role of mTORC2 and GATOR2 in FCD still remains to be elucidated. The application of other techniques besides capture sequencing, such as high-resolution arrays, can uncover small structural variants, like gene duplications or deletions and chromosomal rearrangements (Baldassari et al., 2019b). Combining single-cell DNA/RNA sequencing as well as methylome and proteomic analysis might be essential to decipher the etiology of FCDs.

## Author Contributions

JJ-R: study design, search strategy implementation, study selection, data extraction, data analysis, and writing of the manuscript. LMP: study selection, data extraction, data analysis, and revision of the manuscript. JDM: study design, data analysis, and table and image construction. IPR: data analysis, supervision of the work, and revision of the manuscript. OR, FS, AF, and JBM: study design, supervision of the work, and revision of the manuscript. All authors contributed to the article and approved the submitted version.

## Conflict of Interest

The authors declare that the research was conducted in the absence of any commercial or financial relationships that could be construed as a potential conflict of interest.
