# Supplementary material for: Genomic and Epigenetic Advances in Focal Cortical Dysplasia Types I and II: A Scoping Review
Source: Front Neurosci. 2021 Jan 22;14:580357. doi: 10.3389/fnins.2020.580357 (PMC7862327; doi:10.3389/fnins.2020.580357)
Supplement: Supplementary file 1 [file Data_Sheet_1.docx]

Supplementary Material

# Supplementary Tables

| **Table 1. Overview of the search strategy** | | | |
| --- | --- | --- | --- |
| **Database** | **PubMed** | **Embase** | **Web of Science** |
| **Date of search** | 07.05.2020 | 07.05.2020 | 07.05.2020 |
| **Genomic search query** | ("focal cortical dysplasia" OR “focal cortical dysplasias” OR “focal cortical malformation” OR "focal cortical malformation") AND (“somatic mutation” OR "somatic mutations" OR “somatic variant” OR "somatic variants" OR “mosaicism” OR “germline mutation” OR "germline mutations" OR “germline variant” OR "germline variants" OR “mammalian target of rapamycin” OR “MTOR” OR “AKT1” OR “AKT3” OR “PIK3CA” OR “PIK3R2” OR “PTEN” OR “PDPK1” OR “TSC1” OR “TSC2” OR “RHEB” OR “DEPDC5” OR “GATOR1” OR “NPRL2” OR “NPRL3” OR “SLC35A2” OR “PCDH19” OR “IRS1” OR “RAB6B” OR “RALA” OR “HTR6” OR “ZNF337”))) OR ((("Cortical Dysplasia-Focal Epilepsy Syndrome" [Supplementary Concept] OR "Focal cortical dysplasia of Taylor" [Supplementary Concept])) AND (("Germ-Line Mutation"[Mesh]) OR "Mosaicism"[Mesh]) OR ( "Mechanistic Target of Rapamycin Complex 2"[Mesh] OR "Mechanistic Target of Rapamycin Complex 1"[Mesh] OR "TOR Serine-Threonine Kinases"[Mesh] )) OR "AKT1 protein, human" [Supplementary Concept]) OR "AKT3 protein, human" [Supplementary Concept]) OR "PIK3CA protein, human" [Supplementary Concept])) OR "phosphoinositol-3 kinase regulatory subunit 2, human" [Supplementary Concept])) OR "PTEN Phosphohydrolase"[Mesh]) OR "PDPK1 protein, human" [Supplementary Concept]) OR "TSC1 protein, human" [Supplementary Concept]) OR "TSC2 protein, human" [Supplementary Concept]) OR "RHEB protein, human" [Supplementary Concept]) OR "NPRL2 protein, human" [Supplementary Concept]) OR "UDP-galactose translocator" [Supplementary Concept]) OR "PCDH19 protein, human" [Supplementary Concept]) OR "IRS1 protein, human" [Supplementary Concept]) OR "RALA protein, human" [Supplementary Concept])) | (('focal cortical dysplasia':ti,ab,kw OR 'focal cortical dysplasias':ti,ab,kw OR 'focal cortical malformation':ti,ab,kw OR 'focal cortical malformations':ti,ab,kw) AND ('somatic mutation':ti,ab,kw OR 'somatic mutations':ti,ab,kw OR 'somatic variant':ti,ab,kw OR 'somatic variants':ti,ab,kw OR mosaicism:ti,ab,kw OR 'germline mutation':ti,ab,kw OR 'germline mutations':ti,ab,kw OR 'germline variants':ti,ab,kw OR 'mammalian target of rapamycin':ti,ab,kw OR mtor:ti,ab,kw OR akt1:ti,ab,kw OR akt3:ti,ab,kw OR pik3ca:ti,ab,kw OR pik3r2:ti,ab,kw OR pten:ti,ab,kw OR pdpk1:ti,ab,kw OR tsc1:ti,ab,kw OR tsc2:ti,ab,kw OR rheb:ti,ab,kw OR depdc5:ti,ab,kw OR gator1:ti,ab,kw OR nprl2:ti,ab,kw OR nprl3:ti,ab,kw OR slc35a2:ti,ab,kw OR pcdh19:ti,ab,kw OR 'irs1or rab6b':ti,ab,kw OR rala:ti,ab,kw OR htr6:ti,ab,kw OR znf337:ti,ab,kw)) OR ('cortical dysplasia'/exp AND ('somatic mutation'/exp OR 'mosaicism'/exp OR 'germline mutation'/exp OR 'mammalian target of rapamycin'/exp OR 'mtor gene'/exp OR 'akt1 gene'/exp OR 'akt3 gene'/exp OR 'pik3ca gene'/exp OR 'pik3r2 gene'/exp OR 'pten gene'/exp OR 'pdpk1 gene'/exp OR 'tsc1 gene'/exp OR 'tsc2 gene'/exp OR 'rheb gene'/exp OR 'depdc5 gene'/exp OR 'nprl2 gene'/exp OR 'nprl3 gene'/exp OR 'slc35a2 gene'/exp OR 'pcdh19 gene'/exp OR 'irs1 gene'/exp OR 'rala gene'/exp OR 'htr6 gene'/exp)) | TS=((focal cortical dysplasia OR focal cortical dysplasias OR focal cortical malformation OR focal cortical malformations) AND (somatic mutation OR somatic mutations OR somatic variant OR somatic variants OR mosaicism OR germline mutation OR germline mutations OR germline variant OR germline variants OR mammalian target of rapamycin OR MTOR OR AKT1 OR AKT3 OR PIK3CA OR PIK3R2 OR PTEN OR PDPK1 OR TSC1 OR TSC2 OR RHEB OR DEPDC5 OR GATOR1 OR NPRL2 OR NPRL3 OR SLC35A2 OR PCDH19 OR IRS1 OR RAB6B OR RALA OR HTR6 OR ZNF337)) |
| **Number of results** | 171 | 374 | 312 |
| **Epigenetic search query** | ("focal cortical dysplasia" OR “focal cortical dysplasias” OR “focal cortical malformation” OR "focal cortical malformation") AND (epigenetics OR “epigenetic regulation” OR “gene expression” OR “DNA methylation” OR “RNA sequence” OR “non-coding RNA” OR microRNA OR miRNA OR “long non-coding RNA” OR lncRNA OR “Histone acetylation” OR “Histone deacetylation” OR “Histone methylation”))) OR ((("Focal cortical dysplasia of Taylor" [Supplementary Concept] OR "Cortical Dysplasia-Focal Epilepsy Syndrome" [Supplementary Concept])) AND ((((((((("Epigenomics"[Mesh] OR "Epigenesis, Genetic"[Mesh]) OR "Gene Expression Regulation"[Mesh]) OR "DNA Methylation"[Mesh]) OR "Sequence Analysis, RNA"[Mesh]) OR "RNA, Untranslated"[Mesh]) OR "MicroRNAs"[Mesh]) OR "RNA, Long Noncoding"[Mesh]) OR "Histone Acetyltransferases"[Mesh]) OR "Histone Deacetylases"[Mesh]) | (('focal cortical dysplasia':ti,ab,kw OR 'focal cortical dysplasias':ti,ab,kw OR 'focal cortical malformation':ti,ab,kw OR 'focal cortical malformations':ti,ab,kw) AND (epigenetics:ti,ab,kw OR 'epigenetic regulation':ti,ab,kw OR 'gene expression':ti,ab,kw OR 'dna methylation':ti,ab,kw OR 'rna sequence':ti,ab,kw OR 'non-coding rna':ti,ab,kw OR microrna:ti,ab,kw OR mirna:ti,ab,kw OR 'long non-coding rna':ti,ab,kw OR lncrna:ti,ab,kw OR 'histone acetylation':ti,ab,kw OR 'histone deacetylation':ti,ab,kw OR 'histone methylation':ti,ab,kw)) OR ('cortical dysplasia'/exp AND ('epigenetics'/exp OR 'epigenetic regulation'/exp OR 'gene expression'/exp OR 'dna methylation'/exp OR 'rna sequence'/exp OR 'untranslated rna'/exp OR 'microrna'/exp OR 'long untranslated rna'/exp OR 'histone acetylation'/exp OR 'histone deacetylation'/exp OR 'histone methylation'/exp)) | TS=((focal cortical dysplasia OR focal cortical dysplasias OR focal cortical malformation OR focal cortical malformations) AND (epigenetics OR epigenetic regulation OR gene expression OR DNA methylation OR RNA sequence OR non-coding RNA OR microRNA OR miRNA OR long non-coding RNA OR lncRNA OR Histone acetylation OR Histone deacetylation OR Histone methylation)) |
| **Number of results** | 78 | 212 | 163 |
| **Total** | 220 | 527 | 407 |

| **Table 2. Summary of genomic findings in FCD** | | | | | | | | | |
| --- | --- | --- | --- | --- | --- | --- | --- | --- | --- |
| **NP diagnosis** | **Gene** | **cDNA variant (protein change)** | **Mutation information** | **No. of subjects meeting inclusion criteria** | **% of affected individuals** | **Brain mosaic rate (%)** | **Samples for DNA extraction** | **Methods** | **Reference**  **(author, journal, year of publication)** |
| **FCD I** | *DEPDC5* | c.715C>T (p.Arg239*)  +  c.1264C>T (p.Arg422*)  c.1264C>T (p.Arg422*) | Germline (inherited)  +  Somatic  Germline | 4 (with NP diagnosis of FCD I and II, 2 with brain specimen genetic analysis) | - | - | Peripheral blood,  FFPE brain tissue | Deep targeted seq, Sanger seq | Baulac et al., Ann Neurol., 2015 |
|  | *PCDH19* | duplication of exons 3,4,5a | Germline (de novo) | 2 (with epilepsy surgery, both FCDI and II) | - | - | Peripheral blood | Sanger seq, MLPA | Kurian et al., Dev Med Child Neurol., 2018 |
| **FCD Ia** | *STXBP1* | c.1631G>T (p.Gly544Val) | Germline (de novo) | 1 (with epilepsy surgery) | - | - | Peripheral blood | Direct seq,  multiplex amplicon quantification | Weckhuysen et al.,Epilepsia, 2013 |
|  | *SCN1A* | c.317C>T (p.Ser106Phe) | Germline (inherited) | 2 (with epilepsy surgery, both FCDI and II) | - | - | Peripheral blood | PCR+ Cycle Seq | Barba et al., Epilepsia, 2014 |
|  | *NPRL2* | c.68_69delCT (p.Ile23Asnfs*6) | Germline | 2 (with epilepsy surgery, both FCDI and II) | - | - | Peripheral blood | Targeted gene panel seq | Weckhuysen et al., Epilepsia, 2016 |
|  | *KCNT1* | c.2800G>A (p.Ala934Thr) | Germline (de novo) | 3 (with epilepsy surgery) | - | - | Peripheral blood | Targeted gene panel seq | Rubboli et al., Ann Clin Transl Neurol., 2019 |
|  | *SLC35A2* | c.339_340insCTC (p.Leu113dup)  c.634_635del (p.Ser212Leufs*9) | All somatic | 2 (with NP diagnosis) | - | 2.4-14.3 | Paired peripheral blood and brain tissue | High-depth exome and ultra-high-depth targeted gene seq; ddPCR, Sanger seq | Winawer et al., Ann Neurol.,2018 |
| **FCD Ib** | *AKT3* | duplication of chrom. 1q21.1-q44 | Somatic | 1 (with NP diagnosis) | - | - | Peripheral blood, saliva, brain tissue | Array-CGH; microsatellite analysis; qPCR analysis | Conti et al., Clin Genet., 2015 |
|  | *STXBP1* | deletion of exons 3–4  +  mosaic for heterozygous and homozygous mutations in the dysplastic tissue | Germline (de novo)  +  Somatic | 1 (with epilepsy surgery) | - | - | Peripheral blood,  FF brain tissue | Chromosomal microarray, targeted NGS, ddPCR | Uddin et al., Neurol Genet., 2017 |
|  | *KCNT1* | c.2849G>A (p.Arg950Gln)  c.2386T>C (p.Tyr796His) | Germline (inherited) | 3 (with epilepsy surgery) | - | - | Peripheral blood | Targeted gene panel seq | Rubboli et al., Ann Clin Transl Neurol., 2019 |
| **FCD Ic** | *DEPDC5* | c.1310delA (p.Asn437Metfs*21) | Germline (inherited) | 6 (with NP diagnosis of FCD I and II) | - | - | Peripheral blood | Sanger seq, qPCR | Baldassari et al., Genet Med., 2019 |
| **FCD IIa** | *MTOR* | c.7280T>C (p.Leu2427Pro);  c.6577C>T (p.Arg2193Cys);  c.1871G>A (p.Arg624His);  c.5126G>A (p.Arg1709His) | All somatic | 77 (with FCD II) | 6.49 (5 out of 77)  15.6 (12 out of 77) for both FCD IIa and IIb | 1.26-12.63 | Paired peripheral blood/saliva and  FF/FFPE brain tissue | Deep WES, site-specific amplicon seq, hybrid capture, PCR amplicon seq | Lim et al., Nat Med., 2015 |
|  |  | c.4487T>G (p.Trp1456Gly) | Somatic | 1 | - | 8.3 | Peripheral blood, brain tissue | WES | Leventer et al., Neurology, 2015 |
|  |  | c.4379T>C (p.Leu1460Pro)  c.6644C>T (p.Ser2215Phe)  c.6644C>A (p.Ser2215Tyr) | All somatic | 7 (with FCDII) | 57.1 (4 out of 7) | 1.2-8.6 | Paired peripheral blood/saliva and  brain tissue | WES | Mirzaa et al., JAMA Neurol., 2016 |
|  |  | c.6644C>T (p.Ser2215Phe)  c.6644C>A (p.Ser2215Tyr) | All somatic | 16 (with FCD II) | 12.5 (2 out of 16)  37.5 (6 out of 16) for both FCD IIa and IIb | 1.13-1.6 | Paired peripheral blood and FF brain tissue | Deep-targeted gene panel seq, ddPCR | Moller et al., Neurol Genet., 2016 |
|  |  | c.4376C>A (p.Ala1459Asp)  c.4379 T>C (p.Leu1460Pro)  c.6644C>T (p.Ser2215Phe)  c.6644C>A (p.Ser2215Tyr)  c.7498A>T (p.Ile2500Phe) | All somatic | 54 (with FCD II) | 20.4 (11 out of 54)  35.2 (19 out of 54) for both IIa and IIb  Total 59.3 (32 out of 54) | 0.25-15.67 | Paired peripheral blood and FF brain tissue | Hybrid capture seq targeting, ultra-deep site-specific amplicon seq, ddPCR, Sanger seq, LCM | Baldassari et al., Acta Neuropathol., 2019 |
|  |  | c.1871G>A (p.Arg624His)  c.5126G>A (p.Arg1709His)  c.5930C>A (p.Thr1977Lys)  c.7280T>C (p.Leu2427Pro)  c.6577C>T (p.Arg2193Cys)  c.6644C>T (p.Ser2215Phe)  c.4379T>C (p.Leu1460Pro)  c.6400C>T (p.Arg2134Trp) | All somatic | 107 (with FCD II) | 9.3 (10 out of 107)  19.6 (21 out of 107) for both IIa and IIb  Total 31.8 (34 out of 107) | 0.98-12.6 | Paired and unpaired peripheral blood /saliva and FF/FFPE brain samples. | Targeted gene hybrid capture seq panel, site-specific amplicon seq | Sim et al., Acta Neuropathol., 2019 |
|  | *PIK3CA* | c.3139C>T (p.His1047Arg) | Somatic | 19 (with FCD II) | 5.3 (1 out of 19) | 4.7 | FF brain tissue | Multiplex targeted seq (MIP capture technology),  Sanger seq, qPCR | Jansen et al., Brain, 2015 |
|  | *AKT1* | c.1099C>T (p.Arg367Cys) | - | 11 (with FCDII) | 9.1 (1 out of 11)  Total 18.2 (2 out of 11) | - | FF brain tissue | Targeted gene panel seq, Sanger seq | Kobow et al., Epilepsia, 2019 |
|  | *AKT3* | c.49G>A (p.Glu17Lys) | Somatic | 54 (with FCD II) | 3.7 (2 out of 54)  Total 59.3 (32 out of 54) | 1.1-2.3 | Paired peripheral blood and FF brain tissue | Hybrid capture seq targeting, ultra-deep site-specific amplicon seq, ddPCR, Sanger seq, LCM | Baldassari et al., Acta Neuropathol., 2019 |
|  | *TSC1* | c.610C>T (p.Arg204Cys)  c.64C>T (p.Arg22Trp) | All somatic | 40 (with FCD II) | 7.5 (3 out of 40)  12.5 (5 out of 40) for both FCD IIa and IIb | 1.0-2.0 | Paired saliva and FFPE brain tissue | Targeted deep hybrid capture and PCR amplicon seq, ddPCR | Lim et al., Am J Hum Genet., 2017 |
|  |  | c.610C>T (p.Arg204Cys)  c.64C>T (p.Arg22Trp) | All somatic | 107 (with FCD II) | 2.8 (3 out of 107)  5.6 (6 out of 107) for both FCD IIa and IIb  Total 31.8 (34 out of 107) | 1-2 | Paired and unpaired peripheral blood /saliva and FF/FFPE brain samples. | Targeted gene hybrid capture seq panel, site-specific amplicon seq | Sim et al., Acta Neuropathol., 2019 |
|  | *TSC 2* | c.4639G>A (p.Val1547Ile) | Somatic | 40 (with FCD II) | 0.93 (1 out of 107)  12.5 (5 out of 40) for both FCD IIa and IIb | 1.55 | Paired saliva and FFPE brain tissue | Targeted deep hybrid capture, PCR-based amplicon seq,  ddPCR, site-specific amplicon seq | Lim et al., Am J Hum Genet., 2017 |
|  |  | c.4639G>A (p.Val1547Ile) | Somatic | 107 (with FCD II) | 0.9 (1 out of 107)  1.8 (2 out of 107) for both FCD IIa and IIb  Total 31.8 (34 out of 107) | 1.6 | Paired and unpaired peripheral blood /saliva and FF/FFPE brain samples. | Targeted gene hybrid capture seq panel, site-specific amplicon seq | Sim et al., Acta Neuropathol., 2019 |
|  | *RHEB* | c.104A>T,105C>A (p.Tyr35Leu) | Doublet somatic | 8 (with FCD II) | 12.5 (1 out of 8) | 5.8-6 | Paired peripheral blood and FF brain tissue | WES, targeted seq, Sanger seq | Zhao et al., Exp Mol Med., 2019 |
|  | *DEPDC5* | c.484-1G>A (p.?)  c.1759C>T (p.Arg587*) | Germline mosaic (inherited)  Germline (inherited) | 4  (with NP diagnosis of FCD I and II, 2 with brain specimen genetic analysis) | - | - | Peripheral blood,  FFPE brain tissue | Deep targeted seq, Sanger seq | Baulac et al., Ann Neurol., 2015 |
|  |  | c.1663C>T (p.Arg555*) | Germline (inherited) | 2 | - | - | Peripheral blood | WES, Sanger seq | Scerri et al., Ann Clin Transl Neurol.,2015 |
|  |  | c.842A>T (p.Tyr281Phe) | Germline (inherited) | 1 (with NP diagnosis) | - | - | Peripheral blood | Targeted resequencing (MIP) | Carvill et al., Neurol Genet., 2015 |
|  |  | c.2620C>T (p.Arg874*) | Germline | 52 (with FCD) | 1.9 (1 out of 52)  Total 9.6 (5 out of 52) | - | Peripheral blood,  saliva, brain tissue | Targeted deep seq; Sanger seq, ddPCR; subcloning; single-cell analysis | D'Gama et al.,Cell Rep., 2017 |
|  |  | c.856C>T (p.Arg286*)  +  c.865C>T (p.Gln289*) | Germline (inherited)  +  2-hit somatic | 10 (with FCD II) | 10 (1 out of 10) | -  10 | Paired peripheral blood,  FFPE brain tissue | Deep targeted capture seq, Sanger seq, ultra-deep amplicon seq | Ribierre et al., J Clin Investig., 2018 |
|  |  | c.2390delA (p.Gln797Argfs*18)  +  c.3994C > T (p.Arg1332*) | Germline (de novo)  +  2-hit somatic | 1 | - | -  3.9 | Peripheral blood,  FF / FFPE brain tissue | Deep seq, ddPCR, stereology, LCM | Lee et al., Ann Clin Transl Neurol., 2019 |
|  |  | c.279+1G>A (p.?)  c.1264C>T (p.Arg422*) | Germline (inherited)  Germline (inherited) | 6 (with NP diagnosis of FCD I and II) | - | - | Peripheral blood | Sanger seq, qPCR | Baldassari et al., Genet Med., 2019 |
|  | *DEPDC5* | c.856C >T (p.Arg286*)  +  c.865C>T (p.Gln289*)  c.279 + 1G >A (p.?)  c.715C>T (p.Arg239*)  c.3021 + 1G >A (p.?)  c.4151_4152insC (p.Glu1385fs) | Germline  +  2-hit somatic  Germline  Germline  Germline + somatic LOH  Germline | 54 (with FCD II) | 9.25 (5 out of 54)  Total 59.3 (32 out of 54) | -  10  - | Paired peripheral blood and FF brain tissue | Hybrid capture seq targeting, ultra-deep site-specific amplicon seq, ddPCR, Sanger seq, LCM | Baldassari et al., Acta Neuropathol., 2019 |
|  |  | c.1114C>T (p.Gln372*)  c.3639G>A (p.Trp1213*)  c.3802C>T (p.Arg1268*)  c.3406A>T (p.Arg1136*) | All germline | 107 (with FCD II) | 3.7 (4 of 107)  4.6 (5 out of 107) for both FCD IIa and IIb  Total 31.8 (34 out of 107) | - | Paired and unpaired peripheral blood /saliva and FF/FFPE brain samples. | Targeted gene hybrid capture seq panel, site-specific amplicon seq | Sim et al., Acta Neuropathol., 2019 |
|  |  | c.483 + 1G>A | Germline | 11 (with FCDII) | 9.1 (1 out of 11)  Total 18.2 (2 out of 11) | - | Peripheral blood, FF brain tissue | Targeted gene panel seq, Sanger seq | Kobow et al., Epilepsia, 2019 |
|  |  | c.3225_3226insGAAAGGT (p.Asp1075fs) | Germline | 5 (with FCD II) | 20 (1 out of 5) | - | FFPE brain tissue | Targeted NGS, microdissection | Ying et al., Epileptic Disord., 2019 |
|  | *NPRL2* | c.562C>T (p.Gln188*) | Germline | 52 (with FCD) | 1.9 (1 out of 52)  Total 9.6 (5 out of 52) | - | Peripheral blood,  saliva, brain tissue | Targeted deep seq, Sanger seq, ddPCR, subcloning, single-cell analysis | D'Gama et al.,Cell Rep., 2017 |
|  |  | c.100C>T (p.Arg34*)  c.683+1G>C (p.?) | Germline (inherited)  Germline | 6 (with NP diagnosis of FCD I and II) | - | - | Peripheral blood | Sanger seq, qPCR | Baldassari et al., Genet Med., 2019 |
|  | *NPRL3* | c.1375_1376dupAC (p.Ser460Profs*20)  c.1352-4delACAGinsTGACCCATCC  c.275G>A (p.Arg92Gln) | All germline | 47 (with FCD II) | 8.5 (4 out of 47) | - | Peripheral blood and brain tissue | WES, Sanger seq | Sim et al., Ann Neurol., 2016 |
|  |  | c.1270C>T (p.Arg424*) | Germline | 2 (with epilepsy surgery, both FCDI and II) | - | - | Peripheral blood | Targeted gene panel seq | Weckhuysen et al., Epilepsia, 2016 |
|  | *DEPTOR* | c.338T>A (p.Leu113His) | Germline (inherited) | 2 | - | - | Peripheral blood | WES, Sanger seq | Scerri et al., Ann Clin Transl Neurol.,2015 |
|  | *SCN1A* | c.2584C>G (p.Arg862Gly) | Germline (de novo) | 2 (with epilepsy surgery, both FCD I and II) | - | - | Peripheral blood | PCR+ Cycle Seq | Barba et al., Epilepsia, 2014 |
|  | *PCDH19* | c.696T>A (p.Asn232Lys) | Germline (de novo) | 2 (with epilepsy surgery, both FCD I and II) | - | - | Peripheral blood | Sanger seq, MLPA | Kurian et al., Dev Med Child Neurol., 2018 |
|  | *RAB6B* | c.383C>T (p.Thr128Met) | Somatic | 17 (with FCD II) | 5.9 (1 out of 17)  Total 35.3  (6 out of 17) | 2.71 | Paired peripheral blood and FF brain tissue | WES, site-specific amplicon seq | Zhang et al., Epilepsia, 2020 |
| **FCD IIb** | *MTOR* | c.6644C>T (p.Ser2215Phe);  c.7280T>A (p.Leu2427Gln);  c.5930C>A (p.Thr1977Lys);  c.4348T>G (p.Tyr1450Asp);  c.4447T>C (p.Cys1483Arg) | All somatic | 77 (with FCD II) | 9.1 (7 out of 77)  15.6  (12 out of 77) for both FCD IIa and IIb | 1.51-9.77 | Paired peripheral blood/saliva and  FF/FFPE brain tissue | Deep WES, site-specific amplicon seq, hybrid capture, PCR amplicon seq | Lim et al., Nat Med., 2015 |
|  |  | c.6644C>A (p.Ser2215Tyr)  c.4376C>A (p.Ala1459Asp)  c.4379T>C (p.Leu1460Pro)  c.6644C>T (p.Ser2215Phe) | All somatic | 13 (with FCD IIb) | 46.2 (6 out of 13) | 1.11-9.31 | Paired peripheral blood/saliva and  FF brain tissue | WES, targeted amplicon seq, ddPCR | Nakashima et al., Ann Neurol., 2015 |
|  |  | c.6644C>T (p.Ser2215Phe)  c.6644C>A (p.Ser2215Tyr)  c.4379T>C (p.Leu1460Pro)  c.4375G>T (p.Ala1459Ser) | All somatic | 16 (with FCD II) | 25 (4 out of 16)  37.5 (6 out of 16) for both FCD IIa and IIb | 2.41-6.31 | Paired peripheral blood and FF brain tissue | Deep-targeted gene panel seq, ddPCR | Moller et al., Neurol Genet., 2016 |
|  |  | c.4379T>C (p.Leu1460Pro)  c.4447T>C (p.Cys1483Arg) | All somatic | 52 (with FCD) | 3.8 (2 out of 52)  Total 9.6 (5 out of 52) | 2.3-10.6 | Peripheral blood,  saliva, brain tissue | Targeted deep seq; Sanger seq, ddPCR; subcloning; single-cell analysis | D'Gama et al.,Cell Rep., 2017 |
|  |  | c.4379T>C (p.Leu1460Pro) | Somatic | 7 | 14.3 (1 out of 7)  Total 28.5 (2 out of 7) | 1.8 | Peripheral blood,  FF / FFPE brain tissue | Deep NGS customized gene panel | Avansini et al., Ann Neurol., 2018 |
|  |  | c.4376C>A (p.Ala1459Asp)  c.4379 T>C (p.Leu1460Pro)  c.5930C>A (p.Thr1977Lys)  c.6644C>T (p.Ser2215Phe)  c.6644C>A (p.Ser2215Tyr) | All somatic | 54 (with FCD II) | 14.8 (8 out of 54)  35.2 (19 out of 54) for both IIa and IIb  Total 59.3 (32 out of 54 | 0.74-8.43 | Paired peripheral blood and FF brain tissue | Hybrid capture seq targeting, ultra-deep site-specific amplicon seq, ddPCR, Sanger seq, LCM | Baldassari et al., Acta Neuropathol., 2019 |
|  |  | c.6644C>T (p.Ser2215Phe)  c.7280T>A (p.Leu2427Gln)  c.5930C>A (p.Thr1977Lys)  c.4348T>G (p.Tyr1450Asp)  c.4447T>C (p.Cys1483Arg)  c.4366T>G(p.Trp1456Gly)  c.4376C>A (p.Ala1459Asp)  c.6644C>A (p.Ser2215Tyr) | All somatic | 107 (with FCD II) | 10.3 (11 out of 107)  19.6 (21 out of 107) for both IIa and IIb  Total 31.8 (34 out of 107) | 1.7-6.9 | Paired and unpaired peripheral blood /saliva and FF/FFPE brain samples. | Targeted gene hybrid capture seq panel, site-specific amplicon seq | Sim et al., Acta Neuropathol., 2019 |
|  |  | c.5930C>A (p.Thr1977Lys); | Somatic | 17 (with FCD II) | 5.9 (1 out of 17)  Total 35.3  (6 out of 17) | 4.66 | Paired peripheral blood and FF brain tissue | WES, site-specific amplicon seq | Zhang et al., Epilepsia, 2020 |
|  | *PIK3C3* | c.760A>G (p.Lys254Glu) | Germline | 14 (with FCD) | 7.1 (1 out of 14)  Total 35.7 (5 out of 14) | - | Peripheral blood, saliva, FF brain tissue | WES; MIP seq; Sanger seq; ddPCR; subcloning | D'Gama et al., Ann Neurol., 2015 |
|  | *PIK3C2B* | c.3011C>G (p.Ala1004Gly) | Germline | 14 (with FCD) | 7.1 (1 out of 14)  Total 35.7 (5 out of 14) | - | Peripheral blood, saliva, FF brain tissue | WES; MIP seq; Sanger seq; ddPCR; subcloning | D'Gama et al., Ann Neurol., 2015 |
|  | *PTEN* | c.834C>G (p.Phe278Leu) | Somatic | 34 (with FCD IIb) | 2.94 (1 out of 34) | - | FFPE dysplastic and adjacent normal brain tissue, peripheral blood (control) | Single-strand conformation polymorphism and sequence analysis, LCM | Schick et al., Acta Neuropathol., 2006 |
|  | *AKT1* | C.349_351del(p.Glu117del) | Somatic | 7 (with FCD) | 14.3 (1 out of 7)  Total 28.5 (2 out of 7) | 2.2 | Peripheral blood,  FF/FFPE brain tissue | Deep NGS customized gene panel | Avansini et al., Ann Neurol., 2018 |
|  | *TSC1* | c.453G>A (p.Glu78Lys)  c.549 G>A (p.Ala110Thr)  c.2415C>T (p.His732Tyr) | - | 48 (with FCD IIb) | 2.3-35  2.3 (1 out of 44)  2.3 (1 out of 44)  35 (14 out of 40) | - | FFPE dysplastic and adjacent normal brain tissue, peripheral blood (control) | Single-strand conformation polymorphism and sequence analysis, LCM | Becker et al., Ann Neurol., 2002 |
|  |  | c.64C>T (p.Arg22Trp) | Somatic | 40 (with FCD II) | 2.5 (1 out of 40)  12.5 (5 out of 40) for both FCD IIa and IIb | 1.37 | Paired saliva and FFPE brain tissue | Targeted deep hybrid capture, PCR-based amplicon seq,  ddPCR, site-specific amplicon seq | Lim et al., Am J Hum Genet., 2017 |
|  |  | c.163C>T (p.Gln55*) | Somatic | 52 (with FCD) | 1.9 (1 out of 52)  Total 9.6 (5 out of 52) | 5.1-6.7 | Peripheral blood,  saliva, brain tissue | Targeted deep seq; Sanger seq, ddPCR; subcloning; single-cell analysis | D'Gama et al.,Cell Rep., 2017 |
|  |  | c.1525C >T (p.Arg509*)  c.1907_1908delAG (p.Glu636fs*51) | All somatic | 54 (with FCD II) | 3.7 (2 out of 54)  Total 59.3 (32 out of 54) | 3.4-4.2 | Paired peripheral blood and FF brain tissue | Hybrid capture seq targeting, ultra-deep site-specific amplicon seq, ddPCR, Sanger seq, LCM | Baldassari et al., Acta Neuropathol., 2019 |
|  |  | c.64C>T (p.Arg22Trp)  c.2074C>T (p.Arg692*)  c.1525C>T (p.Arg509*) | All somatic | 107 (with FCD II) | 2.8 (3 out of 107)  5.6 (6 out of 107) for both FCD IIa and IIb  Total 31.8 (34 out of 107) | 1.04-3.9 | Paired and unpaired peripheral blood /saliva and FF/FFPE brain samples. | Targeted gene hybrid capture seq panel, site-specific amplicon seq | Sim et al., Acta Neuropathol., 2019 |
|  | *TSC2* | c.3781G>A (p.Ala1261Thr) | Somatic | 7 (with FCD II) | 14.3 (1 out of 7)  Total 28.5 (2 out of 7) | 1.7 | Peripheral blood,  FF/FFPE brain tissue | Deep NGS customized gene panel | Avansini et al., Ann Neurol., 2018 |
|  |  | c.5228G >A (p.Arg1743Gln)  c.2380C>T (p.Gln794*) | All somatic | 54 (with FCD II) | 3.7 (2 out of 54)  Total 59.3 (32 out of 54) | 1.4-2.15 | Paired peripheral blood and FF brain tissue | Hybrid capture seq targeting, ultra-deep site-specific amplicon seq, ddPCR, Sanger seq, LCM | Baldassari et al., Acta Neuropathol., 2019 |
|  |  | c.1372C>T (p.Arg458*) | Somatic | 107 (with FCD II) | 0.9 (1 out of 107)  1.8 (2 out of 107) for both FCD IIa and IIb  Total 31.8 (34 out of 107) | 3.24 | Paired and unpaired peripheral blood /saliva and FF/FFPE brain samples. | Targeted gene hybrid capture seq panel, site-specific amplicon seq | Sim et al., Acta Neuropathol., 2019 |
|  |  | c.5227C>T (p.Arg1743Trp) | Somatic | 17 (with FCD II) | 5.9 (1 out of 17)  Total 35.3 (6 out of 17) | 4.82 | Paired peripheral blood and FF brain tissue | WES, site-specific amplicon seq | Zhang et al., Epilepsia, 2020 |
|  | *RHEB* | c.119A >T (p.Glu40Val) | Somatic | 54 (with FCD II) | 1.8 (1 out of 54)  Total 59.3 (32 out of 54) | 8.4-8.8 | Paired peripheral blood and FF brain tissue | Hybrid capture seq targeting, ultra-deep site-specific amplicon seq, ddPCR, Sanger seq, LCM | Baldassari et al., Acta Neuropathol., 2019 |
|  | *DEPDC5* | c.783_786delTGAG (p.Asn261Lysfs*11)  c.624+1G>A (p.?)  c.1218-18_1218-15delTGTT (p.?)  c.1355C>T (p.Ala452Val) | All germline | 14 (with FCD) | 21.4 (3 out of 14)  Total 35.7 (5 out of 14) | - | Peripheral blood, saliva, FF brain tissue | WES; MIPS; Sanger seq; ddPCR; subcloning | D'Gama et al., Ann Neurol., 2015 |
|  |  | c.1400_1401insGG (pPhe467Leufs*51) | Germline (inherited) | 6 (with NP diagnosis of FCD I and II) | - | - | Peripheral blood | Sanger seq, qPCR | Baldassari et al., Genet Med., 2019 |
|  |  | c.4521_4522delAA (p.Thr1508fs)  c.4162_4169dupGTACTCTT (p.Phe1399fs) | Germline  +  2-hit somatic | 107 (with FCD II) | 0.93 (1 of 107)  4.6 (5 out of 107) for both FCD IIa and IIb  Total 31.8 (34 out of 107) | -  4.5 | Paired and unpaired peripheral blood /saliva and FF/FFPE brain samples. | Targeted gene hybrid capture seq panel, site-specific amplicon seq | Sim et al., Acta Neuropathol., 2019 |
|  | *IRS1* | c.1791dupG (p.His598Ala fs*13) | Somatic | 17 (with FCD II) | 5.9 (1 out of 17)  Total 35.3 (6 out of 17) | 3.16 | Paired peripheral blood and FF brain tissue | WES, site-specific amplicon seq | Zhang et al., Epilepsia, 2020 |
|  | *ZNF337* | c.692_693del (p.Thr231Arg fs*45) | Somatic | 17 (with FCD II) | 5.9 (1 out of 17)  Total 35.3 (6 out of 17) | 1.84 | Paired peripheral blood and FF brain tissue | WES, site-specific amplicon seq | Zhang et al., Epilepsia, 2020 |
|  | *HTR6* | c.469G>A (p.Ala157Thr) | Somatic | 17 (with FCD II) | 5.9 (1 out of 17)  Total 35.3 (6 out of 17) | 1.29 | Paired peripheral blood and FF brain tissue | WES, site-specific amplicon seq | Zhang et al., Epilepsia, 2020 |
|  | *RALA* | c.482G>A (p.Arg161Gln) | Somatic | 17 (with FCD II) | 5.9 (1 out of 17)  Total 35.3 (6 out of 17) | 5.50 | Paired peripheral blood and FF brain tissue | WES, site-specific amplicon seq | Zhang et al., Epilepsia, 2020 |
| Abbreviations: | NP: neuropathological; Chrom.: chromosome; WES: Whole-Exome Sequencing; Seq: sequencing; ddPCR: droplet digital Polymerase Chain Reaction; MLPA: Multiplex Ligation-dependent Probe Amplification; NGS: Next Generation Sequencing; FF: Fresh frozen; FFPE: Formalin-fixed paraffin-embedded; array-CGH: array Comparative Genomic Hybridization; qPCR: quantitative Polymerase Chain Reaction; MIP: Molecular Inversion Probe; LCM: Laser-Capture Microdissection | | | | | | | | |

| **Table 3. Summary of the epigenetic mechanisms in FCD** | | | | | | | | |
| --- | --- | --- | --- | --- | --- | --- | --- | --- |
| **Epigenetic mechanism** | **FCD subtype** | **Samples** | **No. of patients** | **Type of controls** | **No. of Controls** | **Main findings** | **Methods** | **Reference**  **(author, journal, year of publication)** |
| **DNA methylation** | IIa, IIb | FF brain tissue | 10 | Non-epilepsy controls (autopsy) | 10 | Differential DNA methylation profile compared to controls; inverse correlation between methylation status and gene expression; upregulation of DNMT3α. | Genome-wide  CpG-DNA methylation  microarray analysis, RNA Seq, qRT-PCR. | Dixit et al., Sci Rep., 2018 |
|  | Ia, IIa, IIb | FF brain tissue | 15 | TLE;  Non-epilepsy controls (autopsy + surgical samples) | 11 | Differentially methylated regions  distinguished  FCD subtypes from TLE and nonepileptic controls. | Genome‐wide Methyl-Seq, mRNA-Seq. | Kobow et al., Epilepsia, 2019 |
| **MicroRNA** | IIa, IIb | FF and FFPE brain tissue | 9 | Autopsy | 5 | 23 microRNAs differentially expressed compared to controls;  differentially expression of miR-17~92 cluster; comparison of FCD IIa and IIb, with six types of miRNA differentially expressed between the two groups. | Microarray analysis, qRT-PCR. | Dogini et al, J Epilepsy Clin Neurophysiol., 2012 |
|  | Ia, Ib, IIa, IIb | FF brain tissue | 15 | Normal cortical tissue from surgery for deep seated lesions | 4 | Upregulation of hsa-miR-21 and hsa-miR-155;  in comparison with the normal brain tissue, more significantly different miRNAs were detected in FCD type II than in type I; miRNA (hsa-miR-877) differentially expressed between FCD type I and type II. | Microarray analysis, qRT-PCR | Lee et al.,Epilepsy Res., 2014 |
|  | IIb | FFPE brain tissue | 5 | Matched normal tissue  adjacent to the lesion. | 5 | 24 microRNAs were differentially expressed, 19 upregulated, and 5 downregulated. | Microarray analysis, qRT-PCR | Li et al., J Child Neurol., 2016 |
|  | Ib, IIa, IIb | FF brain tissue  Serum | 9 | Non-epilepsy controls (normal cortical tissue from surgery for hypertensive cerebral hemorrhage) | 8 | Upregulation of hsa-miR-4521 in dysplastic brain tissue and serum of FCD patients. | Microarray analysis, qRT-PCR | Wang et al., Neurochem Res., 2016 |
|  | Ib, IIa, IIb | FF brain tissue  Plasma | 9 | Non-epilepsy controls (normal cortical tissue from surgery for hypertensive cerebral hemorrhage) | 8 | Upregulation of hsa-miR-323a-5p in dysplastic brain tissue and plasma of FCD patients. | Microarray analysis, qRT-PCR | Che et al.,Genet Testing Mol Biomarkers, 2017 |
|  | IIa, IIb | FF and FFPE brain tissue | 16 | TLE;  Non-epilepsy controls (autopsy) | 28 | Downregulation of hsa-let-7f, hsa-miR-31, and hsa-miR34a; upregulation of NEUROG2; NEUROG2  regulation by hsa-miR-34a interaction with its 5’-UTR region;  NEUROG2 expression in balloon cells and dysmorphic neurons. | Microarray analysis, qRT-PCR, luciferase reporter assays, in situ hybridization. | Avansini et al., Ann Neurol., 2018 |
| Abbreviations: | qRT-PCR: quantitative Real-Time Polymerase Chain Reaction; FF: Fresh frozen; FFPE: Formalin-fixed paraffin-embedded; TLE: Temporal Lobe Epilepsy; RNA: Ribonucleic acid | | | | | | | |
